# Supplementary material for: GWAS of random glucose in 476,326 individuals provide insights into diabetes pathophysiology, complications and treatment stratification
Source: Nat Genet. 2023 Sep 7;55(9):1448–61. doi: 10.1038/s41588-023-01462-3 (PMC10484788; doi:10.1038/s41588-023-01462-3)
Supplement: Supplementary file 1 — Supplementary Note. [file 41588_2023_1462_MOESM1_ESM.pdf]

nature genetics

Article

https://doi.org/10.1038/s41588-023-01462-3

GWAS of random glucose in 476,326 individuals provide insights into diabetes pathophysiology, complications and treatment stratification

In the format provided by the authors and unedited

Supplementary Information

GWAS of random glucose in 476,326 individuals provide insights into diabetes pathophysiology, complications and treatment stratification.

Lagou V, Jiang L, Ulrich A et al.

SUPPLEMENTARY NOTE

RG model selection

We examined the distributions of untransformed and natural logarithmic transformed RG in the first set of six available cohorts. We observed that RG was approximately normally distributed after natural log transformation. We then determined the variables that could have a significant effect on RG by fitting several regression models using naturally log-transformed RG as the outcome with age, sex, BMI and time since last meal as predictors. Modelling of RG revealed significant effects ($P<0.05$) of age, sex, BMI and time since last meal (accounted for as T, T^2 and T^3) in these cohorts (**Supplementary Table 2**). Compared to RG models without T, inclusion of T, T^2 and T^3 increased the proportion of variance explained in the range of 1-6%. Thus, inclusion of this covariate is potentially equivalent to 1-6% increase in study sample size.

Genotyping and quality control

Commercial genome-wide arrays and the Metabochip[1] were used by individual studies for genotyping. Studies with genome-wide arrays undertook imputation of missing genotypes using at least the HapMap 2 CEU reference panel via MACH v1.0[2], IMPUTE v0.3.1/v1.0.0/v2.3.2/v4.1.2[3,4], or Minimac2[5] software (**Supplementary Table 1**). For each study, samples reflecting duplicates, low call rate, gender mismatch, or population outliers were removed. Low-quality SNPs were excluded by the following criteria: call rate <0.95,

minor allele frequency (MAF) <0.01, minor allele count <10, Hardy-Weinberg *P*-value $<10^{-4}$. GWAS were performed with PLINK v1.07, SNPTEST v1.1.5/v2.5.1, EMMAX beta-07Mar2010, LMEKIN v1.8 (R package), Merlin v1.1.2, STATA v11, and ProbABEL v0.4.3 (**Supplementary Table 1**).

GWAS in the UKBB

For the GWAS of the UKBB data we excluded non-white non-European individuals and those with discrepancies in genotyped and reported sex. For the RG definition, we used the same criteria as in the other studies described above. To control for population structure, we adjusted the analyses for six first principal components. The GWAS was performed using the BOLT-LMM v2.3 software[6,7] restricting the analyses to variants with MAF>1% and imputation quality>0.4. Additionally, we performed rare variant analysis across the genome on UKBB data for variants with MAF<1% and imputation quality>0.4.

RG meta-analysis

A locus was considered novel for if it contained only novel RG signals. A signal was considered novel if there were no previously established signals for glycaemic traits[8] within +/- 400 kb of the RG signal in LD ($r^2 \geq 0.01$) with this signal (the look up for glycaemic traits was done on the [19] of June 2023). Additional signals were excluded from the novel loci count. However, these are annotated in **Supplementary Table 3**. All signals, including rare, cross-ancestry and sex-dimorphic, were included in the count and those significant in multiple analyses were counted only once. A signal was considered established for T2D if a lead or additional signal was located within +/- 400 kb of a previously reported association.[8-10]

Conditional analysis

For validation and comparison with the GCTA conditional analysis, we have performed direct conditional analyses to replicate the signals based on AS20 and AST20, respectively, using BOLT-LMM v2.3 (with the same settings as described in **Methods**) with all the lead signals fitted as covariates. We checked for additional distinct signals by using a region-wide threshold of $P \leq 1.0 \times 10^{-5}$ for statistical significance after clumping. We further conducted a 2nd round of conditional analysis with BOLT-LMM v2.3 by fitting all the lead signals and additional signals from the 1st round of conditional analysis as covariates, to check for remaining significant signals.

Whole-exome sequencing

A full release of WES data from the UK Biobank Exome Sequencing Consortium for 469,835 UKBB participants were used to validate RG effects from imputed array data at rare variants. In total, 316,789 UKBB participants of European ancestry had both WES (PLINK binary format) and RG data available and met the inclusion criteria for the RG GWAS. Whole exome sequencing of UKBB samples was done using a modified IDTxGen Exome Research Panel v1.0 (www.idtdna.com). Samples whose reported sex differed from the genetically-determined sex, those with high heterozygosity rates, low sequence coverage and duplicated samples were excluded[11]. Raw sequencing data was analysed in the DNAnexus platform[12]. Variant calling to identify both single nucleotide variants (SNVs) and INDELs was done using the WeCall variant caller (https://github.com/Genomicsplc/wecall). In total, there were ~10 million variants which included 8,086,176 single nucleotide variants (SNVs), 370,958 INDELs and 1,596,984 multi-allelic variants[13]. Majority of the SNVs were coding variants (85%) which included 25.3% synonymous, 53.8% missense and 5.4% predicted loss-of-function

variations[13]. 98% of these variants had 0.001%<MAF<1%. Out of 792 rare variants associated with RG above genome-wide significance in the HRC-imputed UKBB data, 18 were available and nine were validated by the UKBB WES data (**Supplementary Table 9**). Each of 18 rare variants (available in the UKBB WES data) were tested separately for their effect on RG by fitting the AST20 RG linear regression model including the six first principal components using the software PLINK v1.90. We considered a rare variant validated if it had the same direction of effect on RG and reached a nominal threshold of significance ($P \leq 0.05$). Furthermore, we tested the nine validated variants for their independence from the 119 common lead variants in direct conditional analyses by using the 119 variants as covariates in the association analysis. Out of the 7 variants, all remained significant at both, regional ($P<10^{-5}$) and genome-wide ($P<5.0\times10^{-8}$) thresholds (**Supplementary Table 3**). The UKBB WES data were also used for estimating the effects of 16 coding SNVs in *GLP1R* available from the set of 19 SNVs (**Supplementary Table 11**), as sensitivity analysis. These SNVs were tested for their effect on RG by using the same model and software as for the rare variant validation.

GLP-1R pharmacological analysis

Reagents

Custom peptides were purchased from Wuxi Apptec and were at least 95% pure. SNAP-Surface probes were purchased from New England Biolabs. BG-S-S-649[14] was provided by New England Biolabs on a collaborative basis. Furimazine was obtained from Promega.

Plasmids and cell line generation

Wild-type and variant GLP-1R expression plasmids, termed pcDNA5-$SNAP_f$-GLP-1R-SmBiT, were generated by Genewiz to the following design[15]: a fast-labelling $SNAP_f$ tag and upstream

signal peptide based on that of the 5-HT_{3A} receptor (MDSYLLMWGLLTFIMVPGCQA), plus C-terminal SmBiT tag, were appended to the codon-optimised wild-type or variant human GLP-1R sequence (without the endogenous N-terminal signal peptide, which would lead to cleavage of the N-terminal SNAP-tag; accordingly, known missense variants in the signal peptide region were not included), and inserted into the pcDNA5/FRT/TO expression vector. These constructs allow bio-orthogonal labelling of expressed GLP-1R using SNAP-labelling probes and monitoring of cytosolic protein interactions made to GLP-1R. Constructs were used either for transient transfection (for a screen of 197 variants using a single agonist responses) or to generate stable cell lines (for more in-depth characterisation using full concentration-responses). To obtain cell populations with inducible expression of SNAP-GLP-1R-SmBiT from a single genomic locus, Flp-In™ T-REx™ 293 cells[16] (Thermo Fisher) were co-transfected with pOG44 (Thermo Fisher) and wild-type or variant pcDNA5-$SNAP_f$-GLP-1R-SmBiT in a 9:1 ratio, followed by selection with 100 µg/ml hygromycin. The resulting cell lines were maintained in DMEM supplemented with 10% foetal bovine serum (FBS) and 1% penicillin/streptomycin.

High content imaging-based GLP-1R internalisation assay

Where stable cell lines were used (i.e. **Figures 2a-b**), wild-type or variant T-REx-SNAP-GLP-1R-SmBiT cells were seeded (10,000/well) in poly-D-lysine-coated black, clear-bottom 96-well plates, in complete medium supplemented with tetracycline (0.2 µg/ml) for 24 hours before the assay. Medium was removed and cells labelled with 0.5 µM BG-S-S-649 (a gift from New England Biolabs) in complete medium for 20 min at 37°C. Agonists were then applied in serum-free medium at the indicated dose for a 30-min stimulation period to induce GLP-1R internalisation. Further details on this assay are given in the Supplementary Note. A series of

concentrations spanning the response range were used. Cells were then washed with HBSS, followed by a 5-min treatment ± 100 mM sodium 2-mercaptoethanesulfonate (Mesna) in alkaline TNE buffer (pH 8.6) to cleave residual surface BG-S-S-649 without affecting that internalised whilst bound to SNAP-GLP-1R. After re-washing, the plate was imaged using a 0.75 numerical aperture 20x phase contrast objective, with 9 fields-of-view (FOVs) per well acquired for both transmitted phase contrast and epifluorescence. Flat-field correction of epifluorescence images was performed using BaSiC v1[17] and cell segmentation was performed using PHANTAST v1[18] for the phase contrast image. To determine specific GLP-1R labelling, cell-free background per image was determined from the segmented epifluorescence image and subtracted from the mean fluorescence intensity from the cell-containing regions. Ligand induced effects were determined by subtracting the signal from vehicle-treated cells exposed to Mesna. Responses were normalised to signal from labelled, untreated cells (i.e. total surface labelling) within the same assay. GLP-1R surface expression levels were also obtained from these assays from wells not treated with GLP-1RA or Mesna. For transient transfection assays (i.e. **Figure 2j**), the assay was performed similarly but with the following changes: 1) HEK293T cells in poly-D-lysine-coated black clear-bottom 96-well plates were transfected using Lipofectamine 2000 with 0.1 µg/well wild-type or variant SNAP-GLP-1R-SmBiT and the assay performed 24 hours later; 2) the plate was imaged as above both prior to and after ligand treatment (+subsequent Mesna cleavage); 3) surface labelling quantification was obtained from the pre-treatment read, and total internalised receptor was obtained from the post-treatment read.

Credible set analysis

Based on the method adopted from[19], for each signal, we defined a ± 1Mb region around it and extracted all the variants that are in linkage disequilibrium with it ($r^2 \geq 0.01$). For the extracted variants, we then calculated the approximate Bayes factor (ABF) by setting the prior variance to 0.04, and further computed their posterior probability of association (PPA) by dividing every ABF by the sum of ABF for all variants within the region. Finally, for each signal, we defined its 99% credible set by sorting all the variants based on descending PPA and extracting variants until a sum of PPA of 0.99 was achieved.

DEPICT analysis

Tissue and cell types enriched for prioritised genes were computed on normalised expression data comprised of 209 tissues and cell types from 37,427 Affymetrix U133 Plus 2.0 Array[20]. We used 500 permutations for bias adjustment and 50 replications for false discovery rate estimation in our analysis in order to calculate empirical *P*-values and false discovery rate cutoffs for prioritised tissues.

CELLECT analysis

Expression values were normalised by using a scaling factor of 10k transcripts. The normalised values were transformed by taking log(x+1), followed by filtering out infrequently expressed genes, and keeping only those mouse transcripts with 1-1 mapping to human genes in Ensembl v.91. This data was supplied to CELLEX to compute a cumulative expression specificity metric (ESμ) of every gene for each Tabula muris cell type by combining four different expression specificity measures[21]. ESμ values were converted to stratified LD-score regression (S-LDSC) annotations using the 1000 Genomes Project SNPs and mapping each SNP

to the strongest ESμ value within 100kb. Cell types were prioritised by S-LDSC on the basis of ESμ-derived annotations and GWAS summary statistics from the current RG meta-analysis.

Mendelian Randomisation analysis

MR can provide estimates of the effect of modifiable exposures on an outcome (e.g. disease) unaffected by classical confounding or reverse causation, whenever randomised clinical trials are not feasible. Valid causal effect estimates can only be derived if the following core MR assumptions hold: i) the genetic instrument(s) are associated with the exposure, ii) the genetic instrument(s) are not associated with the confounders of the exposure-outcome relationship, iii) the genetic instrument(s) influence the outcome only via the exposure.

Instrument selection: Independent (established by conditional analyses for both RG and the lung function phenotypes) genome-wide significant ($P<5.0\times10^{-8}$) variants were selected as genetic instruments. In total, 133 independent variants were defined for RG by the current study, 424 T2D signals were reported for Europeans by Vujkovic *et al.*[10] and 130/162 independent signals were reported by Shrine *et al.*[22] for FVC and FEV1, respectively. We looked for proxy variants with a minimum r^2 of 0.8 where the instrumental variant was not present in the outcome data. Palindromic variants with minor allele frequency larger than 45% were excluded to avoid uncertainty when harmonizing effects to the exposure-increasing allele. After filtering, 136 variants were used to instrument RG and 413 variants were available as T2D instruments. For FVC, 125 and 115 variants could be used as instruments in the RG and T2D MR analyses, respectively. For FEV1, 157 and 140 variants served as instruments in the RG and T2D MR analyses, respectively.

The MR-Egger causal estimate is valid as long as the pleiotropic effects of the instruments are independently distributed from their genetic associations with the risk factor. If the MR-Egger

intercept term is close to zero, then the MR-Egger causal estimate will be close to the IVW estimate. However, even if the two causal estimates are similar, inferences from the two methods can differ if the MR-Egger estimate is imprecise. In such cases, the MR-Egger method provides no additional evidence for a causal effect, but it does not contradict evidence for a causal effect from a conventional (in this case IVW) MR analysis either[23]. Causal effect estimates of RG on lung function are given in units of standard deviation per mmol/L (natural log transformed) RG increase. Similarly, causal effect estimates of lung function measures on RG are expressed in natural log transformed mmol/L RG per standard deviation increase in lung function. We used the STROBE-MR reporting guideline for MR studies to facilitate the readers` evaluation of our results[24].

Polygenic risk score analysis

The PRS for an individual is the summation of the effect (trait-increasing) alleles weighted by the effect size of the SNP taken from the base data. The SNPs in the base data are clumped so that they are largely independent of each other and thus their effects can be summed. To assess predictive power, PRS for RG, T2D and FG were regressed onto the phenotypes of interest (i.e. RG, T2D and HbA1c) providing the coefficient of determination (R^2) as an estimate for the correlation between the phenotype and the PRS in the VUMC cohort. All models were adjusted for age, four principal components, sex and the cohort-specific batch effect. Since the optimal *P*-value threshold for including SNPs in the PRS is unknown a priori, PRS are calculated over a range of thresholds and regressed onto the phenotype of interest, optimising prediction accordingly. The R^2 estimates for each trait were derived by subtracting the R^2 from the null model (*Phenotype ~ sex + age + 4 principal components + batch)* from the

R^2 from the full model (*Phenotype ~ PRS + sex + age + 4 principal components + batch*) which contains the PRS at the best predicting P-value threshold.

STUDY-SPECIFIC ACKNOWLEDGEMENTS AND FUNDING

Airwave

The Airwave Health Monitoring Study was funded by the UK Home Office (780- TETRA, 2003-2018) and is currently funded by the MRC and ESRC (MR/R023484/1) with additional funding from the NIHR Imperial College Biomedical Research Centre (BRC) in collaboration with Imperial College NHS Healthcare Trust. We thank all Airwave participants for their contribution to the study.

BRIGHT

This work was funded by the Medical Research Council of Great Britain (grant number: G9521010D). The BRIGHT study is extremely grateful to all the patients who participated in the study and the BRIGHT nursing team. This work formed part of the research themes contributing to the translational research portfolio for the NIHR Barts Cardiovascular Biomedical Research Centre. The funders had no role in study design, data collection and analysis.

Brisbane Adolescent Twin Study / SSAGA-NAG adult cohort

Genotyping and phenotyping were supported by the Australian National Health and Medical Research Council (389891, 389892, 496739), the EU 5th Framework Programme GenomEUtwin Project (QLG2-CT-2002-01254) and the U.S. National Institutes of Health (AA07535, AA13320, AA13321, AA13326, AA14041, DA12854). Participants gave informed consent and the studies were approved by appropriate institutional review boards.

deCODE

We thank participants in deCODE genetic studies whose contribution made this work possible.

EMIL

EMIL-Cohort, a population-based cohort; the study was approved by the ethical committee of the Chamber of Physicians Baden-Württemberg in the year 2002 (Registration Number 133-02; dates 05.09.2002 and 24.09.2002). We thank Silke Rosinger, Simone Claudi-Boehm, Rosina Sing, Sabine Schilling and Angelika Kurkhaus for technical support.

EPIC-Norfolk

The EPIC-Norfolk study (https://doi.org/10.22025/2019.10.105.00004) has received funding from the Medical Research Council (MR/N003284/1 and MC-UU_12015/1) and Cancer Research UK (C864/A14136). The genetics work in the EPIC-Norfolk study was funded by the Medical Research Council (MC_PC_13048). We are grateful to all the participants who have been part of the project and to the many members of the study teams at the University of Cambridge who have enabled this research.

FINRISK87

Support for FUSION was provided by NIH grants R01-DK062370 (to M.B.), R01-DK072193 (to K.L.M.), and intramural project number 1Z01-HG000024 (to F.S.C.). Genome-wide genotyping was conducted by the Johns Hopkins University Genetic Resources Core Facility SNP Center at the Center for Inherited Disease Research (CIDR), with support from CIDR NIH contract no. N01-HG-65403.

Framingham Heart Study

The Framingham Heart Study (FHS) was supported by the National Heart, Lung and Blood Institute's Framingham Heart Study in collaboration with Boston University (Contract Nos. N01-HC-25195 and HHSN268201500001I and 75N92019D00031) and its contract with

Affymetrix, Inc for genotyping services (Contract No. N02-HL-6-4278), and by NIDDK R01DK078616, U01DK078616, UM1DK078616 and NHLBI R01 HL151855.

HUNT2/Tromsø

The Nord-Trøndelag Health Study (the HUNT study) is a collaboration between HUNT Research Centre (Faculty of Medicine, Norwegian University of Science and Technology NTNU), Nord-Trøndelag County Council, Central Norway Health Authority, and the Norwegian Institute of Public Health. University of Tromsø, Norwegian Research Council (project number 185764).

InterAct

We thank all EPIC participants and staff and the InterAct Consortium members for their contributions to the study. The InterAct project received funding from the European Union (Integrated Project LSHM-CT-2006-037197 in the Framework Programme 6 of the European Community). We thank staff from the technical, field epidemiology and data teams of the Medical Research Council Epidemiology Unit in Cambridge, UK, for carrying out sample preparation, DNA provision and quality control, genotyping and data handling work.

KORA F3

The KORA research platform (KORA, Cooperative Research in the Region of Augsburg) was initiated and financed by the Helmholtz Zentrum München–German Research Center for Environment and Health, which is funded by the German Federal Ministry of Education and Research and by the state of Bavaria. Furthermore, part of this work was supported by the German National Genome Research Network (NGFN) and the Munich Center of Health Sciences (MC Health) as part of LMUinnovativ.

PROCARDIS

PROCARDIS was supported by the European Community Sixth Framework Program (LSHM-CT- 2007-037273), AstraZeneca, the British Heart Foundation, the Wellcome Trust (Contract No. 075491/Z/04), the Swedish Research Council, the Knut and Alice Wallenberg Foundation, the Swedish Heart-Lung Foundation, the Torsten and Ragnar Söderberg Foundation, the Strategic Cardiovascular and Diabetes Programs of Karolinska Institutet and Stockholm County Council, the Foundation for Strategic Research and the Stockholm County Council. Ethical permission was granted by the local ethical board for each centre.

Rotterdam Study

The generation and management of GWAS genotype data for the Rotterdam Study is supported by the Netherlands Organisation of Scientific Research NWO Investments (nr. 175.010.2005.011, 911-03-012). This study is funded by the Research Institute for Diseases in the Elderly (014-93-015; RIDE2), the Netherlands Genomics Initiative (NGI)/Netherlands Organisation for Scientific Research (NWO) project nr. 050-060-810. We thank Pascal Arp, Mila Jhamai, Marijn Verkerk, Lizbeth Herrera and Marjolein Peters for their help in creating the GWAS database, and Karol Estrada and Maksim V. Struchalin for their support in creation and analysis of imputed data. The Rotterdam Study is funded by Erasmus Medical Center and Erasmus University, Rotterdam, Netherlands Organization for the Health Research and rs12874929pment (ZonMw), the Research Institute for Diseases in the Elderly (RIDE), the Ministry of Education, Culture and Science, the Ministry for Health, Welfare and Sports, the European Commission (DG XII), and the Municipality of Rotterdam. The authors are grateful to the study participants, the staff from the Rotterdam Study and the participating general practitioners and pharmacists.

The Section of Endocrinology and Investigative Medicine is funded by grants from the MRC, BBSRC, NIHR, and is supported by the NIHR Biomedical Research Centre Funding Scheme. The

views expressed are those of the author(s) and not necessarily those of the any of the funders, the NHS, the NIHR or the Department of Health.

Vanderbilt

The samples and/or dataset(s) used for the analyses described were obtained from Vanderbilt University Medical Center's BioVU which is supported by numerous sources: institutional funding, private agencies, and federal grants. These include the NIH funded Shared Instrumentation Grant S10OD017985 and S10RR025141; and CTSA grants UL1TR002243, UL1TR000445, and UL1RR024975. Genomic data are also supported by investigator-led projects that include U01HG004798, R01NS032830, RC2GM092618, P50GM115305, U01HG006378, U19HL065962, R01HD074711; and additional funding sources listed at https://victr.vumc.org/biovu-funding/". Additional NIH Grants R01 HL146588, R01 HL146588-01S1 and 1R01DK124845-01A1.

REFERENCES

1. Voight, B.F. *et al.* The metabochip, a custom genotyping array for genetic studies of metabolic, cardiovascular, and anthropometric traits. *PLoS Genet* **8**, e1002793 (2012).
2. Li, Y., Willer, C., Sanna, S. & Abecasis, G. Genotype imputation. *Annu Rev Genomics Hum Genet* **10**, 387-406 (2009).
3. Howie, B.N., Donnelly, P. & Marchini, J. A flexible and accurate genotype imputation method for the next generation of genome-wide association studies. *PLoS Genet* **5**, e1000529 (2009).
4. Marchini, J., Howie, B., Myers, S., McVean, G. & Donnelly, P. A new multipoint method for genome-wide association studies by imputation of genotypes. *Nat Genet* **39**, 906-13 (2007).
5. Fuchsberger, C., Abecasis, G.R. & Hinds, D.A. minimac2: faster genotype imputation. *Bioinformatics* **31**, 782-4 (2015).
6. Loh, P.R., Kichaev, G., Gazal, S., Schoech, A.P. & Price, A.L. Mixed-model association for biobank-scale datasets. *Nat Genet* **50**, 906-908 (2018).
7. Loh, P.R. *et al.* Efficient Bayesian mixed-model analysis increases association power in large cohorts. *Nat Genet* **47**, 284-90 (2015).
8. Chen, J. *et al.* The trans-ancestral genomic architecture of glycemic traits. *Nat Genet* **53**, 840-860 (2021).
9. Mahajan, A. *et al.* Identification and functional characterization of G6PC2 coding variants influencing glycemic traits define an effector transcript at the G6PC2-ABCB11 locus. *PLoS Genet* **11**, e1004876 (2015).

10. Vujkovic, M. *et al.* Discovery of 318 new risk loci for type 2 diabetes and related vascular outcomes among 1.4 million participants in a multi-ancestry meta-analysis. *Nat Genet* **52**, 680-691 (2020).
11. Van Hout, C.V. *et al.* Exome sequencing and characterization of 49,960 individuals in the UK Biobank. *Nature* **586**, 749-756 (2020).
12. Reid, J.G. *et al.* Launching genomics into the cloud: deployment of Mercury, a next generation sequence analysis pipeline. *BMC Bioinformatics* **15**, 30 (2014).
13. Szustakowski, J.D. *et al.* Advancing human genetics research and drug discovery through exome sequencing of the UK Biobank. *Nat Genet* **53**, 942-948 (2021).
14. Fang, Z. *et al.* The Influence of Peptide Context on Signaling and Trafficking of Glucagon-like Peptide-1 Receptor Biased Agonists. *ACS Pharmacol Transl Sci* **3**, 345-360 (2020).
15. Fang, Z. *et al.* Ligand-Specific Factors Influencing GLP-1 Receptor Post-Endocytic Trafficking and Degradation in Pancreatic Beta Cells. *Int J Mol Sci* **21**(2020).
16. Ward, R.J., Alvarez-Curto, E. & Milligan, G. Using the Flp-In T-Rex system to regulate GPCR expression. *Methods Mol Biol* **746**, 21-37 (2011).
17. Peng, T. *et al.* A BaSiC tool for background and shading correction of optical microscopy images. *Nat Commun* **8**, 14836 (2017).
18. Jaccard, N. *et al.* Automated method for the rapid and precise estimation of adherent cell culture characteristics from phase contrast microscopy images. *Biotechnol Bioeng* **111**, 504-17 (2014).
19. Wakefield, J. A Bayesian measure of the probability of false discovery in genetic epidemiology studies. *Am J Hum Genet* **81**, 208-27 (2007).
20. Pers, T.H. *et al.* Biological interpretation of genome-wide association studies using predicted gene functions. *Nat Commun* **6**, 5890 (2015).
21. Timshel, P.N., Thompson, J.J. & Pers, T.H. Mapping heritability of obesity by brain cell types. *bioRxiv*, 2020.01.27.920033 (2020).
22. Shrine, N. *et al.* New genetic signals for lung function highlight pathways and chronic obstructive pulmonary disease associations across multiple ancestries. *Nat Genet* **51**, 481-493 (2019).
23. Burgess, S. & Thompson, S.G. Interpreting findings from Mendelian randomization using the MR-Egger method. *Eur J Epidemiol* **32**, 377-389 (2017).
24. Skrivankova, V.W. *et al.* Strengthening the reporting of observational studies in epidemiology using mendelian randomisation (STROBE-MR): explanation and elaboration. *BMJ* **375**, n2233 (2021).
