# Supplementary material for: GWAS of random glucose in 476,326 individuals provide insights into diabetes pathophysiology, complications and treatment stratification
Source: Nat Genet. 2023 Sep 7;55(9):1448–61. doi: 10.1038/s41588-023-01462-3 (PMC10484788; doi:10.1038/s41588-023-01462-3)
Supplement: Supplementary file 2 — Reporting Summary [file 41588_2023_1462_MOESM2_ESM.pdf]

nature portfolio

Corresponding author(s): Prof Inga Prokopenko, Dr Ben Jones, Dr Marika Kaakinen

Last updated by author(s): Jun 20, 2023

Reporting Summary

Nature Portfolio wishes to improve the reproducibility of the work that we publish. This form provides structure for consistency and transparency in reporting. For further information on Nature Portfolio policies, see our Editorial Policies and the Editorial Policy Checklist.

Statistics

For all statistical analyses, confirm that the following items are present in the figure legend, table legend, main text, or Methods section.

n/a	Confirmed	
☐	☒	The exact sample size (*n*) for each experimental group/condition, given as a discrete number and unit of measurement
☐	☒	A statement on whether measurements were taken from distinct samples or whether the same sample was measured repeatedly
☐	☒	The statistical test(s) used AND whether they are one- or two-sided<br>*Only common tests should be described solely by name; describe more complex techniques in the Methods section.*
☐	☒	A description of all covariates tested
☐	☒	A description of any assumptions or corrections, such as tests of normality and adjustment for multiple comparisons
☐	☒	A full description of the statistical parameters including central tendency (e.g. means) or other basic estimates (e.g. regression coefficient) AND variation (e.g. standard deviation) or associated estimates of uncertainty (e.g. confidence intervals)
☐	☒	For null hypothesis testing, the test statistic (e.g. *F*, *t*, *r*) with confidence intervals, effect sizes, degrees of freedom and *P* value noted<br>*Give P values as exact values whenever suitable.*
☒	☐	For Bayesian analysis, information on the choice of priors and Markov chain Monte Carlo settings
☒	☐	For hierarchical and complex designs, identification of the appropriate level for tests and full reporting of outcomes
☐	☒	Estimates of effect sizes (e.g. Cohen's *d*, Pearson's *r*), indicating how they were calculated

Our web collection on statistics for biologists contains articles on many of the points above.

Software and code

Policy information about availability of computer code

Data collection	No software was used for the data collection.
Data analysis	The software and tools used in individual GWAS can be found in Supplementary Table 1 with participating studies characteristics. Software/tools/algorithms included: Minimac2, MACH v1.0, IMPUTE v0.3.1/v1.0.0/v2.3.2/v4.1.2, SNPTEST v1.1.5/v2.5.1, EMMAX vbeta-07Mar2010, LMEKIN v1.8 (R package), Merlin v1.1.2, STATA v11, ProbABEL v0.4.3, BOLT-LMM v2.3, SS-Imp v0.5.5, METAL v2011-03-25, GWAMA v2.1, PLINK v1.07/1.90, GCTA v1.93.0, BaSiC v1, AceMD3 3.3.0, PHANTAST v1, AquaMMapS v1, DEPICT v1_rel 194, CELLECT v1.0.0, CELLEX v1.0.0, MetaXcan (S-PrediXcan) v0.6.10, GARFIELD v2, COLOC2, LDSC v1.0.0, TwoSampleMR v0.5.4 (R package), PRSice v2.2.3, LDlinkR v1.1.2 library, MVMR v0.3 (R package), Prism 8.0 (GraphPad Software), IDTxGen Exome Research Panel v1.0.

For manuscripts utilizing custom algorithms or software that are central to the research but not yet described in published literature, software must be made available to editors and reviewers. We strongly encourage code deposition in a community repository (e.g. GitHub). See the Nature Portfolio guidelines for submitting code & software for further information.

Data

Policy information about availability of data

All manuscripts must include a data availability statement. This statement should provide the following information, where applicable:

- Accession codes, unique identifiers, or web links for publicly available datasets
- A description of any restrictions on data availability
- For clinical datasets or third party data, please ensure that the statement adheres to our policy

Meta-analyses summary statistics for the GWAS presented in this manuscript are available on the MAGIC website (magicinvestigators.org) and through the NHGR1-EBI GWAS Catalog (https://www.ebi.ac.uk/gwas/downloads/summary-statistics, GCP ID: GCP000470). UK Biobank individual-level data can be obtained through a data access application available at https://www.ukbiobank.ac.uk/. In this study we made use of data made available by: 1000 Genomes project https://www.genome.gov/27528684/1000-genomes-project; SNPsnap https://data.broadinstitute.org/mpg/snpsnap/index.html; Tabula Muris https://www.czbiohub.org/tabula-muris/; GTEx Consortium https://gtexportal.org/home/; microbiome GWAS https://mibiogen.gcc.rug.nl/; Human Gut Microbiome Atlas https://www.microbiomeatlas.org; eQTLGen Consortium https://www.eqtlgen.org/; TIGER expression data http://tiger.bsc.es/; LDHub database http://ldsc.broadinstitute.org/ldhub/.

Human research participants

Policy information about studies involving human research participants and Sex and Gender in Research.

Reporting on sex and gender	As a first step, we have fitted several models in the six cohorts, available to us initially for the modeling of RG, i.e. to identify the relevant set of covariates (including sex) as well as the necessary transformation for RG to be used across all the datasets in the GWAS meta-analysis. We then evaluated six different models in GWAS according to covariates included and cut-offs used: 1) age (A) and sex (S), RG<20 mmol/L (AS20), 2) age, sex and BMI (B), RG<20 mmol/L (ASB20), 3) age and sex, RG<11.1 mmol/L (AS11), 4) age, sex and BMI, RG<11.1 mmol/L (ASB11), 5) age, sex, T, T2 and T3, RG<20 mmol/L (AST20) and 6) age, sex, T, T2 and T3 and BMI, RG<20 mmol/L (ASTB20). To evaluate sex-dimorphism in our results, we meta-analyzed the UKBB and the Vanderbilt cohort with the GMAMA software, which provides a 2 degrees of freedom (df) test of association assuming different effect sizes between the sexes. We considered a signal to show evidence of sex-dimorphism if the 2 df test P-value was <5x10-8 and if the sex heterogeneity P-value (1 df) was <0.05.
Population characteristics	Analyses were conducted on non-diabetic females and males of European ancestry and additionally in non-European populations within UKBB (Black, Indian, Pakistani and Chinese). Within each study, individuals were included based on two RG cut-offs: <20 mmol/l (20) to account for the effect of extreme RG values and <11.1 mmol/l, which is an established threshold for T2D diagnosis. Age distribution and percentage for each gender varied between studies. More detailed description of each study collection is provided in Supplementary Table 1, and for the UK Biobank in https://www.ukbiobank.ac.uk/.
Recruitment	The majority of studies are population-based cohorts, case-control or family-based studies with related individuals. Subjects were men and women of European, Black, Indian, Pakistani or Chinese ancestry with no diagnosed diabetes.
Ethics oversight	No ethical approval was required for the study as it is a meta-analysis of summary statistics obtained from studies that each had ethical approval by local research ethics committees and written consent was obtained from all study participants. Further details about each study ethics approval can be found in the references and websites provided in Supplementary Table 1.

Note that full information on the approval of the study protocol must also be provided in the manuscript.

Field-specific reporting

Please select the one below that is the best fit for your research. If you are not sure, read the appropriate sections before making your selection.

☒ Life sciences ☐ Behavioural & social sciences ☐ Ecological, evolutionary & environmental sciences

For a reference copy of the document with all sections, see nature.com/documents/nr-reporting-summary-flat.pdf

Life sciences study design

All studies must disclose on these points even when the disclosure is negative.

Sample size	We aimed to bring together the largest possible sample size for RG with the following collection of samples: (i) 37,239 individuals from 10 European ancestry GWAS imputed up to the HapMap 2 reference panel; (ii) 3,156 individuals from three European ancestry GWAS with Metabochip coverage; (iii) 21,083 individuals from two European ancestry GWAS imputed up to 1000 Genomes reference panel; (iv) 380,432 individuals of white European ancestry from the UKBB, and; (v) 16,983 individuals from the Vanderbilt cohort imputed to the HRC panel Non-European UKBB populations included in the analyses had a sample size of at least 1,500 individuals. These were Black (N=7,644), Indian (N=5,660), Pakistani (N=1,747) and Chinese (N=1,503). Therefore, exact sample size was not predetermined. Maximum sample size was achieved by including all cohorts with RG available.

Data exclusions	Individuals were excluded from the analysis, if they had a diagnosis of T2D or were on diabetes treatment (oral or insulin). Individual studies applied further sample exclusions, including pregnancy, fasting plasma glucose equal to or greater than 7 mmol/l in a separate visit, when available, and having Type 1 Diabetes. Detailed descriptions of study-specific RG measurements are given in Supplementary Table 1. Low-quality SNPs were excluded by the following criteria: call rate <0.95, minor allele frequency (MAF) <0.01, minor allele count <10, Hardy-Weinberg P-value <10–4. After imputation of the GWAS meta-analysis summary statistics, imputed SNPs up to 1000 Genomes reference panel with imputation quality score < 0.7 were excluded. For the GWAS of the UKBB data, non-white non-European individuals and those with discrepancies in genoped and reported sex were excluded. Furthermore, in the UKBB GWAS, variants with MAF<=1% and imputation quality<=0.4 were excluded.
Replication	We have assessed the robustness of our RG meta-analysis findings by comparing the direction of effect of 133 signals, detected in the European subset between the UK Biobank (UKBB, 83.8% of the total study size) and other RG contributing studies grouped together. We further extended the check between UKBB results and meta-analysis of other RG contributing cohorts together with fasting glucose GWAS excluding overlapping cohorts (roughly 1/3 of UKBB sample size). Results from these comparisons are presented in Supplementary Table 3. Additionally, we have selected a list of additional distinct signals by carrying out approximate conditional analysis with GCTA and direct conditional analyses on UKBB genotypes with BOLT-LMM. To ensure pharmacological assay results were reproducible, all assays were repeated at least 4 times. The exact number of biological replicates is provided in the relevant supplementary table, along with the relevant measure of dispersion.
Randomization	This study meta-analyzed existing data. Therefore, there were no experimental groups and no randomization was required.
Blinding	GWAS is a hypothesis-free approach, so in each study contributing to the meta-analysis, researchers assessing glycemic traits, such as random glucose, were blinded to the genotypes that are associated with these outcomes.

Reporting for specific materials, systems and methods

We require information from authors about some types of materials, experimental systems and methods used in many studies. Here, indicate whether each material, system or method listed is relevant to your study. If you are not sure if a list item applies to your research, read the appropriate section before selecting a response.

Materials & experimental systems

n/a	Involved in the study
☒	☐ Antibodies
☐	☒ Eukaryotic cell lines
☒	☐ Palaeontology and archaeology
☒	☐ Animals and other organisms
☒	☐ Clinical data
☒	☐ Dual use research of concern

Methods

n/a	Involved in the study
☒	☐ ChIP-seq
☒	☐ Flow cytometry
☒	☐ MRI-based neuroimaging

Eukaryotic cell lines

Policy information about cell lines and Sex and Gender in Research

Cell line source(s)	Flp-In T-REx-293 cells were obtained from Thermo Fisher.
Authentication	The Flp-In T-REx-293 cell line were indirectly authenticated by successful integration of the WT/variant GLP1R insert.
Mycoplasma contamination	Cells tested negative for mycoplasma.
Commonly misidentified lines (See ICLAC register)	*Name any commonly misidentified cell lines used in the study and provide a rationale for their use.*
